# Deep Reinforcement Learning-Based Routing Method for Low Earth Orbit Mega-Constellation Satellite Networks with Service Function Constraints

**DOI:** 10.3390/s25041232

**Published:** 2025-02-18

**Authors:** Yan Chen, Huan Cao, Longhe Wang, Daojin Chen, Zifan Liu, Yiqing Zhou, Jinglin Shi

**Affiliations:** 1 University of Chinese Academy of Sciences, Beijing 100049, China; zhouyiqing@ict.ac.cn (Y.Z.); sjl@ict.ac.cn (J.S.); 2 State Key Laboratory of Processors, Institute of Computing Technology, Chinese Academy of Sciences, Beijing 100190, China; wanglonghe@ict.ac.cn (L.W.); chendaojin@ict.ac.cn (D.C.); liuzifan@ict.ac.cn (Z.L.); 3 Institute of Computing Technology, Chinese Academy of Sciences, Beijing 100190, China; 4 Beijing Key Laboratory of Mobile Computing and Pervasive Device, Beijing 100190, China; 5 Nanjing Mobile Communication and Computing Innovation Institute, Chinese Academy of Sciences, Nanjing 211135, China

**Keywords:** LEO satellite network, routing, service function constraints, graph convolution network, deep reinforcement learning

## Abstract

Low-orbit satellite communication networks have gradually become the research focus of fifth-generation (5G) beyond and sixth generation (6G) networks due to their advantages of wide coverage, large communication capacity, and low terrain influence. However, the low earth orbit mega satellite network (LEO-MSN) also has difficulty in constructing stable traffic transmission paths, network load imbalance and congestion due to the large scale of network nodes, a highly complex topology, and uneven distribution of traffic flow in time and space. In the service-based architecture proposed by 3GPP, the introduction of service function chain (SFC) constraints exacerbates these challenges. Therefore, in this paper, we propose GDRL-SFCR, an end-to-end routing decision method based on graph neural network (GNN) and deep reinforcement learning (DRL) which jointly optimize the end-to-end transmission delay and network load balancing under SFC constraints. Specifically, this method constructs the system model based on the latest NTN low-orbit satellite network end-to-end transmission architecture, taking into account the SFC constraints, transmission delays, and network node loads in the end-to-end traffic transmission, uses a GNN to extract node attributes and dynamic topology features, and uses the DRL method to design specific reward functions to train the model to learn routing policies that satisfy the SFC constraints. The simulation results demonstrate that, compared with graph theory-based methods and reinforcement learning-based methods, GDRL-SFCR can reduce the end-to-end traffic transmission delay by more than 11.3%, reduce the average network load by more than 14.1%, and increase the traffic access success rate and network capacity by more than 19.1% and two times, respectively.

## 1. Introduction

At present, the global commercialization of 5G mobile communication networks is advancing [1,2,3,4,5]; however, due to the constraints of geographic conditions and construction costs, it is impossible to achieve seamless global coverage [6]. 6G non-terrestrial network (NTN) technology [7], which is the convergence of satellite communication networks and 5G, has gradually become the focus of attention in academia and industry [8,9,10,11]. For example, the 3rd Generation Partnership Project (3GPP) proposed four star-earth converged network architecture schemes based on on-planet processing/transparent forwarding with/without relaying [12], and ITU proposed four application scenarios for the convergence of 5G and satellite communications [13]. Compared to medium earth orbit (MEO) and geostationary earth orbit (GEO) satellite networks, LEO-MSN has the advantages of low transmission delay, low propagation loss, high regional capacity, and low manufacturing and launching costs, and it has been considered to be an essential part of next-generation communication networks [14,15,16,17,18].

In recent years, a large number of low-orbit mega-constellation programs, represented by StarLink, OneWeb, and Kuiper, have been proposed. These constellations usually consist of hundreds or thousands of satellites and operate in 200–2000 km near-Earth orbits at speed of 7.2 km/s [19]. Among them, SpaceX’s StarLink constellation has grown the most rapidly, with more than 6300 satellites deployed as of June 2024 [20], providing satellite-accessed internet communication services to more than 2.5 million subscribers in more than 70 countries and territories, with up to 500 Mbps downlink speed, 40 Mbps uplink speed, and 20 ms latency internet access services [21].

As one of the key technologies for LEO-MSN communication, routing significantly impacts traffic transmission delay and quality of service [22]. However, in LEO-MSN, the routing technology still faces the following challenges. Firstly, the massive scale of mega constellations and the nodes with high dynamics make the network topology highly complex, and it is difficult to construct stable traffic transmission paths. In addition, the uneven distribution of the traffic in time and space makes the LEO-MSN face problems such as load imbalance and congestion [23]. With the increasing capacity of on-board load processing, the LEO-MSN network architecture has evolved from transparent payload (TP) mode to regenerative payload (RP) [24] mode. According to the Service-Based Architecture (SBA) proposed by 3 GPP [25], the functions of the access network, transmission network, and core network can be segmented on demand and deployed flexibly on the satellite or the ground, which brings new constraints to routing and transmission strategies. Under the new service architecture, the routing policy must satisfy the Service Function Chain (SFC) constraints [26], i.e., the traffic transmission path from the source node to the target node must pass through the nodes of relevant service functions in order.

In the dynamic topology and resource-constrained environment of LEO-MSN, the introduction of SFC constraints will make satellite routing face more challenges. First, the functional nodes need to centrally process multi-path traffic, resulting in drastic fluctuations in network load, which becomes a key bottleneck for network congestion. In addition, the high-speed movement of satellites causes frequent reconstruction of SFC paths. Traditional routing methods lack global perception of the functional chain status and are difficult to maintain service continuity during link switching, further exacerbating the risk of transmission interruption.

Facing the new large-scale satellite network scenarios and the introduction of SFC constraints, existing satellite routing methods are no longer applicable, which is mainly due to the fact that some of the methods only optimize from a single metric. For example, refs. [27,28,29,30,31] only considers the optimization of path delay, refs. [32,33] only considers the optimization of network load without considering the SFC constraints in the service transmission process, and some of the methods [26,34] consider that the SFC constraints only optimize the path delay, neglecting the load optimization of the whole network.

Inspired by AI-based communication algorithms [35,36], this paper proposes a GDRL-SFCR routing decision method that integrates a GNN and DRL to systematically solve the core challenges caused by SFC constraints in LEO-MSN. For the problem of frequent path throughput due to satellite dynamic topology, a GNN is used to extract the link state and functional attributes of multi-hop neighbors, and dynamic graph embedding is used to achieve adaptive sensing of topology changes. For the multi-objective optimization with SFC constraints, low latency, and load balancing, DRL agents are used to dynamically adjust the weights of the composite reward function, and the competitiveness metrics are automatically balanced in the policy optimization. The main contributions of this paper are summarized as follows.

- A more realistic LEO-MSN model: Compared to [26], we consider more constrained models. Specifically, channel model, network functions such as access network, core network, and end-to-end bidirectional traffics in satellite communication networks as specified in the latest 3GPP NTN protocol are considered.
- A novel routing method: We propose a method, GDRL-SFCR to solve the end-to-end routing problem under SFC constraints. This method uses a GNN to learn the dynamically changing topological features of the LEO-MSN network and trains using DRL to learn routing strategies to make appropriate routing decisions, which can rationally utilize network resources and improve network performance. Specifically, making routing decisions with network load balancing minimizes the end-to-end traffic transmission delay while guaranteeing the SFC constraints are met.
- Performance evaluation: We constructed mega-satellite networks to validate the performance of the proposed algorithms, and to the best of our knowledge, the size of the simulation network we used is the largest among the papers available so far. The simulation results show that the proposed method outperforms other routing methods in terms of network metrics such as average end-to-end path delay, average network load, traffic access success rate, and network capacity.

The remainder of this paper is organized as follows. Section 2 summarizes the relevant literature. Section 3 describes the system model and the routing decision problem under SFC constraints. Next, Section 4 describes the proposed GDRL-SFCR method in detail. Then, Section 5 presents and analyzes the simulation results. Finally, Section 6 summarizes this paper.

## 2. Literature Review

Due to the dynamic topology of networks, the limited resources of satellites, and the imbalanced demands of users, ground routing strategies are deemed unsuitable for satellite networks. Consequently, extensive studies had been conducted in the routing of LEO satellite constellations. In this section, we review the recent works in LEO satellite routing. Existing studies have extensively researched routing in satellite networks, and the mainstream routing algorithms can be divided into two types: static routing and dynamic routing. The static routing algorithm discretizes the network topology in time and space and then makes routing decisions, e.g., the “snapshot”-based routing algorithm [27] and the Contact Graph Routing (CGR) algorithm [28]. However, the static routing algorithm cannot be changed in real time according to network status, resulting in a lack of flexibility and fault tolerance [37]. Dynamic routing algorithms make routing decisions by obtaining real-time state information about the satellite network [29,30,31,32,33,38,39]. For example, time-varying graph-based routing algorithms [29,30] construct a time-varying graph model by jointly considering the transmission volume and the start moment of the traffic, the connectivity opportunities of the links, and the storage resources of the nodes, and based on this, they decide on the paths with the minimum latency. In [38], Zhang et al. proposed a routing algorithm based on the global routing table generated on the ground and supplemented by the local routing table generated by satellite nodes. It updates the local and global routing tables based on network information, reducing the routing calculation overhead. In [31], Wu et al. proposed a status and reputation-adaptive-based dynamic routing (SRADR) algorithm that introduces security attributes expressed in terms of node reputation, which guarantees the security of the path while deciding the path with the shortest delay.

The above algorithms are mainly based on the shortest path, which selects a path with the least forwarding nodes during traffic transmission. However, as the amount of traffic in the network increases, the satellite nodes on the shortest path will carry a large amount of traffic flow, which results in an unbalanced network load. Because of this, a series of load balancing routing algorithms have been proposed. The segmentation routing-based load balancing algorithm [32] dynamically divides the light- and heavy-traffic-load areas, adopting the snapshot routing algorithm in the light-traffic-load areas and adopting the minimum weight path defined by the congestion index as the routing rule in the heavy-traffic-load areas. The QoS routing algorithm considers energy consumption (QER) [33] and adjusts the next-hop forwarding node according to the node cache queue size and remaining energy to control the link traffic. Distributed routing algorithms based on neighbor satellite load status [39] alleviate the network condition of satellite congestion by introducing mechanisms such as adjacent satellite load state updating, load balancing, and route adjudication, obtaining low-latency and low-packet-drop rate paths.

However, the above algorithms do not take into account the SFC constraints for traffic transmission and are not suitable under the new network architecture. There have been few efforts to investigate the routing strategy satisfying SFC constraints in the LEO-MSN. In [33], the joint optimization of NFV deployment and routing strategy in the space–air–ground integrated network was studied without considering the time-varying characteristics of this network. In [26], the routing problem in LEO-MSN was studied by considering the SFC constraints; however, there is a lack of consideration of factors such as end-to-end link, channel modeling, and network node load. In [34], Xu et al. introduce an effective Deep Reinforcement Learning (DRL) approach to effectively tackle the SFC routing problem in LEO-MSN.

Previous studies have explored the routing problem in LEO-MSN from several angles, which has laid a good foundation for the study of routing strategies. However, considering the new network architecture, there is still room for improvement in the current study. It mainly includes the following aspects:

- When designing the algorithms, most of the work focuses on the theoretical models and algorithms proposed, and it lacks the consideration of the evolution of network architectures and protocols, as well as the functional constraints of transmission paths;
- This study only considered inter-satellite links, feeder links, and unidirectional traffic, lacking consideration of user links and end-to-end bidirectional traffic flows. Table 1 shows some existing methods for satellite routing.

## 3. System Model and Problem Formulation

### 3.1. LEO-MSN Model

The LEO-MSN communication network, including satellites, ground stations, user terminals, and remote servers, is considered in this paper. The communication starts with a user terminal and ends with a remote server, and the data packets can be forwarded through ISLs of LEO satellite in space. The LEO-MSN at each moment can be defined as an undirected graph G(t)=(V,E(t)), where *V* and *E* represent the set of vertices and edges in the graph, respectively. Node $v_{i}\in V$ represents a satellite, ground station, user terminal, or remote server. Edge $e_{i,j}\in E$ represents a link between two network nodes *i* and *j* in the network. The position of each satellite is calculated based on the simplified general perturbations 4 (*SGP*4) [40] orbital propagation model with the input parameter TLE (Two-Line Element set). The satellite coordinates are updated with the following formula:

(1) $$r_{i}\left(t\right)=SGP4\left(TLE_{i},t\right),\Delta t=60\;s$$

where $TLE_{i}$ contains the parameters of the orbital half-length axis, the eccentricity, the orbital inclination, and the ascending node declination.

Each LEO satellite can establish an Inter-Satellite Link (ISL) with two satellites in the same orbital plane and two satellites in adjacent orbital planes. Since the spatial distance between the front and rear satellites in the same orbital plane is fixed, the ISL within the orbit is assumed to be stable over the system period. For satellites that are uniformly distributed in the same orbital plane, the distance between two neighboring satellites can be calculated using the following equation:

(2) $$L_{inter}=2\left(R_{earth}+h\right)\cdot sin\left(\frac{\pi }{2N_{L}}\right)$$

Satellites in adjacent orbital planes can be calculated using the following formula:

(3) $$L_{adj}=2\left(R_{earth}+h\right)\cdot sin\left(\frac{\Delta \Omega \cdot \sin\theta }{2}\right)$$

where $R_{earth}$ is the radius of the Earth (in km), and *h* is the height of the satellite orbit. $N_{L}$ is the number of satellites in the same orbital plane, θ is the orbital inclination, and ΔΩ is the difference in longitude of the orbital plane. The relevant terms introduced in this section are listed in Table 2.

According to the NTN networking architecture proposed by 3GPP [25], user terminals need to pass through nodes with different functional protocols when accessing the network for service transmission, specifically including the service functions of the access network, transmission network, and core network. In this paper, we assume that the satellites and ground stations in LEO-SN can deploy different network functions, and each satellite or ground station can provide at most one function. Based on this, the network nodes in LEO-SN can be classified into nodes with processing functions and nodes with transparent forwarding functions [41,42], where the processing function nodes can provide certain functions during service transmission, including two kinds of functions, gNB and NGC (core network functions, including AMF, UPF, etc.), which can provide on-board access and on-board data processing functions, while the satellite with transparent forwarding function nodes only provides transparent forwarding service (TM). We can denote the set of nodes with the processing function and the set of nodes with the transparent forwarding function as $VF=\{v_{i}|v_{i}\in \left\{gNB,NGC\right\}\}$, $VNF=\{v_{i}|v_{i}\in \left\{TM\right\}\}$, respectively, so the set of nodes in LEO-SN can also be denoted as $V=VF\cup VNF\cup V_{Src},V_{Dst}$.

### 3.2. Channel Model

In LEO mega-satellite communication networks, transmission channels can be divided into two main categories:

- Inter-satellite link channel model: In this paper, the inter-satellite link channel model we used is the free-space path loss model. The path loss can be expressed as follows:
  (4) $$FSPL={\left(\frac{4\pi Rf}{c}\right)}^{2}$$
  where *R* is the communication distance, *c* is the speed of light (in m/s), and *f* is the communications center frequency (in Hz) of inter-satellite links. The received power $P_{R}$ can be calculated using the following equation:
  (5) $$P_{R}=P_{Tx}\cdot G_{T}\cdot G_{R}\cdot \frac{c^{2}}{{\left(4\pi Rf\right)}^{2}L}$$
  where $P_{Tx}$ is the transmit power, and $G_{T}$ and $G_{R}$ are the transmit and receive antenna gains, respectively. *L* is the additional system loss. The data rate (in bps) of inter-satellite links can be expressed as follows:
  (6) $$R_{ISL}=B\cdot log_{2}\left(1+\frac{P_{R}}{N_{0}B}\right)$$
  here, $N_{0}$ is the noise power spectral density, and *B* is the channel bandwidth.
- Satellite–ground link channel model: This includes the transmission channel between user terminals and satellites as well as between ground stations and satellites. From the ground node to the satellite node, the wireless signal propagation experiences the ground, the atmosphere, and the space environment, and it needs to consider the influence of various factors such as the ground shadow effect, the multipath effect, the atmospheric propagation loss, the free space propagation loss, etc. In this paper, we refer to the relevant parameters of 3GPP TR 38.811 [24] to establish the satellite–ground link channel model. Specifically, the parameter set of the urban scenario in Table 6.7.2 is used.
  The Path Loss (PL) is composed of the following components:
  (7) $$PL=PL_{b}+PL_{g}+PL_{s}+PL_{e}$$
  where PL is the total path loss (in dB), $PL_{b}$ is the basic path loss (in dB), $PL_{g}$ is the attenuation due to atmospheric gasses (in dB), $PL_{s}$ is the attenuation due to either ionospheric or tropospheric scintillation in dB, and $PL_{e}$ is the building entry loss (in dB).
  The basic path loss in units of dB is modeled as follows:
  (8) $$PL_{b}=FSPL\left(s,f_{c}\right)+SF+CL\left(\alpha ,f_{c}\right)$$
  where $FSPL(s,f_{c})$ is the free space path loss, $CL(\alpha ,f_{c})$ is the clutter loss, and SF is the shadow fading loss represented by a random number generated by the normal distribution, i.e., $SF∼N(0,\sigma^{2})$.
  The SNR can be calculated as follows:
  (9) $$SNR=EIRP-k-PL-B+\frac{G}{T}$$
  where EIRP is the effective isotropic radiated power (in dBW), $\frac{G}{T}$ is the antenna gain-to-noise-temperature (in dB/K), *k* is the Boltzmann constant, equal to −228.6 dBW/K/Hz, PL is the path loss (in dB), and *B* is the channel bandwidth (in dBHz). Although the original unit of each component is different, the unit is unified by standardization in decibel operation. By adding and subtracting decibels, the formula unifies the transmit power, path loss, noise, and receive performance into SNR, which visually reflects the link quality.
  EIRP can be calculated as follows:
  (10) $$EIRP=P_{T}-L_{C}+G_{T}$$
  where $P_{T}$ is the antenna transmit power (in dBW), $L_{C}$ is the cable loss (in dB), and $G_{T}$ is the transmit antenna gain (in dBi).
  Antenna gain-to-noise-temperature $\frac{G}{T}$ can be derived as follows:
  (11) $$\frac{G}{T}=G_{R}-N_{f}-10log_{10}\left(T_{0}+\left(T_{\alpha }-T_{0}\right)10^{-0.1N_{f}}\right)$$
  According to the Shannon formula, the data rate (in bps) of the satellite–ground link can be expressed as follows:
  (12) $$R_{GSL}=Blog_{2}\left(1+SNR\right)$$

### 3.3. End-to-End Traffic Transmission Model with Service Function Constraints

**Figure 1:**
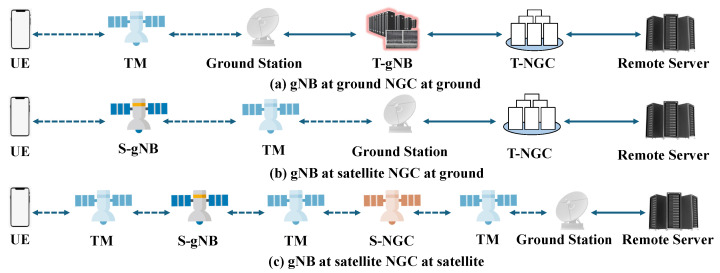
End-to-end traffic transmission model. Dashed arrows indicate wireless links and realized arrows indicate wired links.

According to the NTN networking architecture proposed by 3GPP [25], as shown in Figure 1, the end-to-end traffic transmission modes of the LEO-MSN can be divided into three types: (a) the gNB and the NGC are both on the ground, and the user terminal is relayed to the ground station for processing via the satellite; (b) the gNB is on the satellite and the NGC is on the ground, the user terminal is connected to the satellite node, and the data are transmitted back to the ground; (c) the gNB and the NGC are both on the satellite, and data on the traffic are processed on the satellite without landing. The constraints for completing the end-to-end service transmission in different deployment modes are different, in which the network nodes containing the gNB and NGC functions must be passed through. The path can be expressed as $Path_{Src,Dst}=\left\{V_{Src}\to \cdots \to VF_{i}\cdots \to VF_{FN}\cdots \to V_{Dst}\right\}$, where $VF_{i}$ denotes the ith functional node through which the end-to-end service transmission passes, and FN denotes the total number of functional nodes of the SFC in the path.

The transmission delay of traffic *d* with path $Path_{Src,Dst}$ can be defined as follows:

(13) $$Delay_{d}=\sum_{e_{ij}\in Path_{d}}\frac{Bv_{d}}{R(i,j)}$$

where $Bv_{d}$ represents the data size of traffic *d*, and R(i,j) represents the transmission rate of the link (i,j).

### 3.4. Problem Formulation

Based on the assumptions of the above models, the following constraints can be established:

- Link capacity constraint: At any given moment, the amount of traffic carried on each link must not exceed the link capacity:
  (14) $$0\leq X_{t}^{d}\left(i,j\right)\leq C_{t}\left(i,j\right),\forall e_{i,j}\in E\left(t\right)$$
  where $X_{t}^{d}\left(i,j\right)$ represents the amount of traffic transmitted on link (i,j) at time *t*, and $C_{t}\left(i,j\right)$ represents the capacity of link (i,j) at time *t*.
- Node capacity constraint: The amount of data stored on node *u* cannot exceed the node’s cache capacity limit, i.e.,
  (15) $$0\leq \sum \int_{t}^{t+\Delta t}f_{v,u}\left(t\right)\;dt-\sum \int_{t}^{t+\Delta t}f_{u,w}\left(t\right)\;dt\leq St\left(u\right)$$
  where St(u) is the cache size of the node *u*, and $f_{v,u}\left(t\right)$ and $f_{u,w}\left(t\right)$ denote the flows into and out of node *u* at time *t*, respectively.
- Service function constraint: The traffic with source node Src, destination node Dst, and routing path $Path_{Src,Dst}$ needs to satisfy the service function constraints, i.e.,
  (16) $$Path_{Src,Dst}=\left\{V_{Src}\to \cdots \to VF_{i}\cdots \to VF_{FN}\cdots \to V_{Dst}\right\}$$
  where $VF_{i}$ denotes the ith functional node through which the end-to-end service transmission passes, and FN denotes the total number of functional nodes of the SFC in the path.

In this paper, in order to achieve the joint optimization of network load and path delay for routing under SFC constraints in LEO-MSN, we formulate it as a weighted multi-objective problem and dynamically balance the delay minimization and load balancing through adaptive coefficients. Based on the established model and the constraints mentioned above, the objective function of the routing problem in this paper can be expressed as follows:

(17) $$\begin{array}{cc} \; & \min_{P}\left(\underset{\mathrm{Path}\;\mathrm{Delay}}{\underset{︸}{\theta_{1}\cdot \sum_{Path_{ij}\in Path}Delay_{ij}}}+\underset{\mathrm{Load}\;\mathrm{Balancing}}{\underset{︸}{\theta_{2}\cdot Load}}\right)\\ \; & \begin{array}{cc} \; & s.t.\;\mathrm{Equation}\;(13)–\mathrm{Equation}\;(16) \end{array} \end{array}$$

where $\theta_{1}$ and $\theta_{2}$ are the weight parameters used to balance the influence of the end-to-end delay and network load. $Delay_{i,j}$ denotes the delay of node *i* and *j*, and Load represents the average network load, which can be calculated using the following equation:

(18) $$Load=\frac{1}{N^{\prime }}\sum_{v\in V^{\prime }}^{N^{\prime }}\frac{St_{c}\left(v\right)}{St(v)}$$

where $St_{c}\left(v\right)$ and St(v) denote the used cache capacity and cache capacity of node *v*, respectively; $V^{\prime }$ is the set of satellite nodes and ground station nodes in the network, and $N^{\prime }$ is the number of satellite nodes and ground station nodes. The weighted sum form of Equation (17) aims to transform end-to-end delay minimization and network load balancing into a single-objective problem that can be jointly optimized. Although there may be local conflicts between the two, GDRL-SFCR is able to achieve the co-optimization of the two at the global level by choosing the weights $\theta_{1}$ appropriately.

Since the objective and constraints are linear functions, this class of problems is NP-hard [43], and this optimization problem can be solved using ILP or heuristic algorithms. However, it is non-trivial to use these techniques to model dynamic metrics. Therefore, we propose a graph neural network and deep reinforcement learning-based minimum delay routing method, GDRL-SFCR, which minimizes the delay of the end-to-end transmission path of traffic and reduces the network load while satisfying the SFC constraints.

To systematically address the constraints of link capacity, node capacity, and service function chaining, our proposed GDRL-SFCR framework integrates the following innovations: (a) dynamically extracting network topology features using a GNN and adjusting edge weights in real time according to the available bandwidth of the links to ensure that the routing paths always satisfy the bandwidth constraints; (b) applying penalties to decisions that exceed the node’s memory during DRL intelligence training to ensure that all decision paths satisfy the node capacity constraints; (c) blocking invalid routing options during the exploration phase of DRL intelligence and embedding SFC metadata into the GNN messaging process to ensure that the paths satisfy the functional order constraints.

## 4. Proposed Intelligent Routing Method

In this section, we propose GDRL-SFCR, an end-to-end routing decision method based on a GNN and DRL, which jointly optimizes the end-to-end transmission delay and network load balancing under SFC constraints. In this paper, solving the routing problem with SFC constraints is modeled as a sequential decision-making process that iteratively executes and decides the next hop node for the packet in each state until the destination node is reached. In each iteration, the current state of the network nodes is first obtained and converted into a graph structure. Then, the GNN is applied to extract the feature embedding of the network. Multilayer Perceptron (MLP) is used to obtain the current valid state based on the features of the current node and the neighboring nodes, followed by the use of a decision network to generate the action probability distributions based on the valid states and sampling from them to determine the next hop node.

### 4.1. MDP Formulation

The routing problem in satellite networks can be generally described as follows: obtaining the network state at the current moment by obtaining information about the bandwidth information of the link, the link distance, the satellite network topology, and the node load. Then, decide the next hop for sending packets and pass them to other nodes. After that, each hop node cycles in turn. The action taken by each node is related only to the current state of the network, and the process can be described as a Markov Decision Process (MDP) [44]. We can represent it as a quaternion $\left(S,A,P_{a},R_{a}\right)$, where *S* is the state space that the agent can observe, A denotes the action space associated with the decision, $P_{a}$ is short for $P_{a}\left(S,S^{\prime }\right)$’, which is the probability of changing from state *S* to state $S^{\prime }$ after executing action *a*, and $R_{a}$ is the reward that the agent receives for executing action *a*. In the context of the end-to-end SFC routing problem for satellite networks, we will describe each element of the tuple of the SFC routing decision problem in detail:

(a) State space: Let *S* denote the environment’s finite set of acceptable states. The state consists of six parts: the source node, destination node, current network node, current node load value, number of functional nodes through which the current path passes, and set of neighboring nodes of the current node.
(b) Action space: Define *A* as the set of next-hop satellites that the agent can select at the current moment. Since the number of neighboring nodes is different for different network nodes, in order to unify the size of the action space and minimize the complexity of the action space design, we construct the action space as a one-hot vector with the same size as the node with the highest degree in the network and set the dimension of the action space to dim(G). When the node is $n_{i}$ and when $\dim\left(n_{i}\right)<\dim\left(G\right)$, only the first $\dim(n_{i})$ nodes are considered for sampling from the policy.
(c) Reward: The most important goal of this paper is to achieve load balancing and delay joint optimal routing while satisfying SFC constraints. To achieve this goal, we consider factors such as path delay, network load, and SFC constraints when designing the reward function, which is used to guide the agent to learn the optimization strategy and make correct routing decisions to achieve the goal of this paper. The reward function in this paper combines the following factors:
  - Rewards for network node load $R_{L}$: Load negatively affects latency and network performance, so providing positive rewards for low-loaded nodes and negative rewards for high-loaded nodes is possible.
  - Reward for the number of functional nodes in the current path $R_{F}$: To encourage the agent to choose SFC-compliant paths, we provide positive rewards for paths satisfying SFC constraints and negative rewards for those not, thus guiding the model to optimize step by step during the training process and ultimately satisfy the SFC constraints.
  - Reward for delay between current satellite node and next hop node $R_{T}$: Adding a delay penalty trains the agent to know how to deliver the packet to the destination node with the shortest delay.
  - Reward for other factors: In order to avoid routing loops and enable the model to converge quickly, the occurrence of routing loops is “punished” to reduce the risk of recurring routing loops occurring.

Taking these factors into account, we can define the reward function as follows:

(19) $$\mathrm{Reward}=\left\{\begin{array}{c} -1,u\in \mathrm{Vis}\\ -\left(R_{L}+R_{F}+R_{T}\right),u\notin \mathrm{Vis}\\ -\left(R_{L}+R_{F}+R_{T}\right)+1,u\notin \mathrm{Vis}\;\&\;u=\mathrm{Dst}\;\&\;R_{F}=FN \end{array}\right.$$

where $R_{T}$ denotes the average path delay, $R_{F}$ denotes the number of functional nodes visited, and $R_{L}$ denotes the average network load. Dst denotes the destination node of the current service, *u* denotes the current node, and Vis denotes the set of nodes that have been routed in the current round. If u∈Vis indicates that there is a loop, the reward value is set to the minimum value of −1 to reduce the probability of loop appearance; if u∉Vis, u=Dst, and $R_{F}=FN$, then the destination node is successfully reached, and the reward value is additionally increased by 1 to guide the algorithm to converge in the direction of correctly completing the routing requirements. The rest of the cases are given a normal reward value.

### 4.2. GDRL-SFCR Intelligent Routing Framework

**Figure 2:**
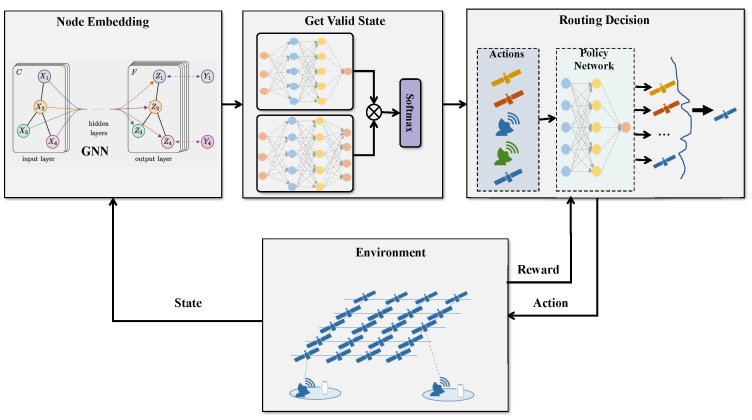
Model architecture of the proposed method.

The model architecture of GDRL-SFCR, illustrated in Figure 2, consists of three components: embedding of the LEO satellite network, obtaining a valid state space, and making the routing decision. In the proposed GDRL-SFCR method, a GNN and DRL are combined to optimize routing decisions. The GNN processes the graph-structured data representing the network topology, capturing essential structural features such as node connections and link properties. Then, a MLP is used to obtain the current valid state based on the features of the current node and the neighboring nodes. These features are then fed into the DRL framework, which learns the optimal policy for routing decisions. We will explain the components in detail.

#### 4.2.1. Embedding of LEO Satellite Network

Graph data employ a data structure that represents entities and relationships by nodes and edges [45]. The connections in a satellite network are modeled as weighted undirected graphs, where nodes (satellites, ground stations, UEs, remote servers) and edges (inter-satellite/ground links) define complex spatial–temporal relationships. Most traditional deep learning models, such as convolutional neural networks (CNNs) and recurrent neural networks (RNNs), cannot be directly applied to graph data due to the lack of capacity to capture geometric features from a graph. This is because traditional models are designed based on the translation invariance or local connectedness of data in Euclidean space. However, graph data do not have the same regular spatial structure as image and text data. The proposal of using a GNN extends the existing neural network mechanism, which can achieve end-to-end processing of graph data [46]. GNNs [47] consist of multi-layer graph convolution, and their essential purpose is to use graph convolutions to extract the spatial features of graph data. They have strong generalization and reasoning capabilities in resource management, end-to-end communication, MIMO detection, and other applications, and they have achieved good results [48,49].

In order to extract the characteristics of the LEO network topology, we utilized a GNN to process the network topology data. It passes messages between the nodes of the graph through an iterative message passing algorithm so that each node can acquire the characteristics of its surrounding neighbors, expanding the node’s perceptual domain, which can help to make more appropriate routing decisions. At each time step *t*, the current state of the LEO satellite network $S_{t}=\left(A;X\right)$ is fed into the GNN to learn a new matrix $H_{t}$.

The forward propagation of the graph convolution is given as follows:

(20) $$H_{t}=\sigma \left({\tilde{D}}^{-\frac{1}{2}}\tilde{A}{\tilde{D}}^{-\frac{1}{2}}XW\right)$$

where σ(•) represents the activation function, and *W* is the trainable parameters. ${\tilde{D}}^{-\frac{1}{2}}\tilde{A}{\tilde{D}}^{-\frac{1}{2}}$ is the approximated graph convolution filter that is similar to the Convolutional Neural Network (CNN). $\tilde{D}$ represents the degree matrix, and ${\tilde{D}}_{ii}=\sum_{j}{\tilde{A}}_{ij}$; $\tilde{A}=A+\wedge$ is the adjacency matrix of the LEO satellite network with added self-connections using a renormalization trick, where ∧ is the identity matrix.

#### 4.2.2. Obtaining State Space and Action Distribution

The above embedding process can be thought of as converting the original feature μ of each network node into an embedding $\mu^{\prime }$ using the GNN. When making a routing decision, we only need to consider selecting an optimal next-hop node based on the states of the current network node and its neighbor nodes. In order to reduce the dimensionality of the state space, we consider using the degree of similarity between the current node and its neighbor nodes as the current effective state space. After applying the GNN, the embedding of the current node $H_{c}$ and the embedding of its neighboring nodes $H_{n}$ are transformed in their dimensions using $MLP_{f}$ and $MLP_{g}$, respectively, after which the inner product of these node embeddings is computed and expanded to be consistent with the dimensions of the states defined earlier in the paper to obtain the current desired effective state, as follows:

(21) $$\begin{array}{c} H_{c}^{\prime }=MLP_{f}\left[H_{c}\right]=W_{f}\cdot H_{c}+b_{f} \end{array}$$

(22) $$\begin{array}{c} H_{n}^{\prime }=MLP_{g}\left[H_{n}\right]=W_{g}\cdot H_{n}+b_{g} \end{array}$$

(23) $$\begin{array}{c} S_{t}^{\prime }=\sum_{j=1}^{N_{f}}\sum_{i=1}^{\dim(G)}H_{c}^{\prime }\left(j\right)\cdot H_{n,i}^{\prime }\left(j\right) \end{array}$$

(24) $$\begin{array}{c} S_{t}=\left[S_{t}^{\prime },0,\cdots ,0\right] \end{array}$$

The above process allows states with different numbers of neighboring nodes to be transformed into valid states with uniform dimensions.

#### 4.2.3. Routing Decision

DRL can solve MDPs by learning long-term strategies that maximize the objective function in an optimization problem. Specifically, the agent learns the optimal policy $\pi_{\omega }\left(a_{t}|S_{t}\right)$ through an iterative process that continuously explores the state and action space and is guided by a reward function. In this paper, our decision-making process involves the agent finding all the neighbor nodes of the current node *v*, generating feature vectors for each case, and inputting them into the GNN. Then, based on the processing results of the GNN, it makes the final routing decision, i.e., the selection of the next-hop forwarding neighbor, using the policy learned from the policy network. In the action selection phase, all path options that violate the SFC order are directly blocked. Based on the objectives of this paper, we designed the corresponding policy network.

For each feasible action $a_{t}\in A_{t}$ at step *t*, the effective state values after the inner product of its state embedding and the state embeddings of its neighboring nodes are sent to the policy network to obtain the index of the distribution in which it is selected in state $S_{t}$ as follows:

(25) $$P\left(a_{t},S_{t}\right)=MLP_{\omega }\left[S_{t}\right]$$

where $MLP_{\omega }$ has three hidden layers and a ReLu activation layer. The probability of selecting each $a_{t}$ is then calculated by applying softmax to all $P(a_{t},S_{t})$:

(26) $$\pi_{\omega }\left(a_{t}|S_{t}\right)=\frac{exp(P\left(a_{t},S_{t}\right))}{\sum_{a_{t}^{\prime }}P\left(a_{t}^{\prime },S_{t}\right)},\forall a_{t}\in A_{t}$$

During training, actions should be sampled according to the policy to explore. There are typically two strategies for solving a given instance using a trained strategy $\pi_{\omega }$, including greedily selecting the actions with a maximum probability and sampling the actions that follow $\pi_{\omega }$ in each state. Unlike the greedy strategy, the random sampling strategy may provide a different solution for each run, which provides an additional possibility to find a better solution. Therefore, this paper adopts the random sampling strategy.

### 4.3. DRL-SFCR Training Algorithm

In the experiments in this paper, GDRL-SFCR adopts a centralized training architecture, where the training nodes are located at the logical center node where the centralized network controller is located. This is mainly because the GNN needs to integrate network topology, node state, and link load information to generate a global graph representation, and a centralized architecture facilitates real-time access to network data. In addition, centralized training ensures that all nodes execute the same policy version and avoids decision conflicts caused by unsynchronized policies in distributed training. The training data are obtained from the full network topology at a fixed period.

This paper uses PPO [50] for training, which employs an actor–critic structure. The actor is the policy network $\pi_{\omega }$, and the critic is the value network $\pi_{\varphi }$ used to predict the value of state st. In this paper, we design the critic as an MLP, which inputs the computed state to obtain $v_{\varphi }\left(s_{t}\right)$. $MLP_{\pi }$ and $MLP_{\varphi }$ have the same structure, with two d1 dimensions and a tanh-activated hidden layer, but with different parameter φ and input dimensions. The routing agent is trained as shown in Algorithm  1 with max_episode training iterations, during which the DRL agent first initializes the environment and obtains the node embeddings of the current network using the GNN, followed by using the MLP to obtain the features of the current node and its surrounding neighboring nodes and calculating their inner product as the input state $S_{t}$. With this information as input, the actor network generates actions $a_{t}$. The environment computes the reward and the next state $S_{t+1}$ based on the current action a1, obtains this information, saves it in the cache queue, uses MSE to compute the loss, and uses the Adam optimizer to optimize the network parameters. The policy is validated in a new environment every five iterations during the training period.
**Algorithm 1** GDRL-SFC training algorithm**Input:** *GNN network, policy network and critic network* **Output:** Some parameters
 1:Initialize actor network and critic network. 2:**for** step=1,2,⋯,max_step **do** 3:    Initialize env, memory buffer; 4:    **while** not Done **do** 5:        Extract node embeddings using GNN; 6:        Get the valid state $s_{t}$ use MLP; 7:        Actor network sample action $a_{t}$; 8:        Calculate the reward $r_{t}$ and receive next state $s_{t+1}$; 9:        Store the current experience in the memory buffer.10:        Update $s_{t}$ = $s_{t+1}$11:    **end while**12:    Sample a batch from the memory buffer.13:    Compute loss L, and optimize the parameters;14:    Update network parameters;15:    **if** Done **then**16:        Break;17:    **end if**18:    **if** tmod5==0 **then**19:        Test the policy;20:    **end if**21:**end forreturn**;

Although GDRL-SFCR performs well in end-to-end routing decision optimization, its computational cost still needs to be considered. (a) GNN message passing overhead: the time complexity of node embedding updates is $O(N^{2})$, where N is the number of network nodes. (b) The agent needs to interact with the environment a great deal in order to converge on the routing decision policy.

Considering the traffic problem that may be caused by the large-scale features of the nodes in the LEO-MSN network, the solution proposed in this paper involves a series of optimizations, which include the following. (a) Reducing the dimensionality of the nodes’ features: in this paper, the GNN adopts 32-dimensional node features and combines them with 8-bit quantization. The single node feature packet is reduced from 512 KB to 256 KB. (b) DRL model lightweighting: the DRL strategy network fuses GNN-MLP features, and it is trained with 16-bit mixed precision with gradient sparsification (threshold θ=1× 10^−5^). The single model update is compressed to 154 KB, and the update frequency is adaptively adjusted to two times per hour. (c) The feature data are transmitted in bulk using the Polar Satellite with a 12-times-over-head window every day (the top of which is not exceeded by the top of the window), and the feature data are transmitted in bulk using the Polar Satellite over the top window batch transmission (8 min each time). Therefore, the total global daily communication amount is 6000 × (256 KB × 4 + 154 KB × 12) = 16.43 GB, and the bandwidth requirement is 16.43 × 8 ÷ (24 × 3600) ≈ 1.55 Mbps. This value is significantly lower than the earth station uplink capacity (>10 Mbps), proving that the scheme is feasible.

## 5. Simulation and Performance Analysis

In this section, we test the performance of the proposed GDRL-SFCR method.

### 5.1. Experiment Setting

#### 5.1.1. Simulation Scenario Setting

The LEO satellite network considered in this paper consists of 6048 LEO satellites, ground stations, and user terminals. Specifically, it is a mega constellation of 6048 satellites constructed with reference to the parameters of StarLink, with 84 constellation orbits, 72 satellites uniformly distributed in each orbit, an orbital inclination of $53^{\circ }$, and an orbital altitude of 550 km [51]. The satellite nodes are randomly assigned the TM, gNB, and NGC functions. For the ground station, StarLink has already completed the deployment of the data [52]. We constructed user terminals of different sizes based on the World Grid Population v4 dataset [53]. The distribution of remote servers uses the same data as the Starlink ground station, and the source node is randomly chosen from one of the user terminals, while the destination node is randomly chosen from the remote servers. The data flow size is in the range of [5, 100] Mb and satisfies the lognormal distribution. In order to verify the performance of the proposed algorithm, we conducted 1000 simulation experiments. The algorithms were implemented in Python (Version 3.13.2). The critical simulation parameters are shown in Table 3.

#### 5.1.2. Model Parameters

In this paper, the convolutional layer of the GNN is set to L=2, and the embedding dimension of the satellite network node is set to d=6. For the MLP, the hidden dimension is set to $d_{h}=64$ and $d_{\pi }=d_{\phi }=4$. For training, the number of training iterations and maximum number of trial sizes were set to max_episode=10,000 and max_step=50, respectively. For the PPO loss function, the coefficients of policy loss (with a clipping ratio of 0.2), value loss, and entropy were set to 1, 0.6, and 0.02, respectively. The PPO optimization period was set to R=3, and the discount factor was set to γ=1. The actor and critic networks used the Adam optimizer to update the parameters, and the learning rate was $lr=10^{-4}$. To solve the problem of long model training time, we used OpenRL [54] to accelerate the training process. All experiments were performed using a machine equipped with an Intel i7-11700K CPU (Intel Corporation, Santa Clara, CA, USA), 32 GB of memory, an Nvidia GeForce RTX 3080 GPU (NVIDIA Corporation, Santa Clara, CA, USA), and an Ubuntu 22.04 64-bit OS.

### 5.2. Methods of Comparison

In order to verify the effectiveness of the proposed method, we compared GDRL-SFCR with other routing methods, including the SFC-constrained augmenting path selection (SFC-APS) [26] algorithm, the DQN-LBR [39] algorithm, and the Deep Q-Routing (DQR) [34] algorithm.

SFC-APS: SFC-APS is SFC routing method based on traditional graph theory; by joining a virtual node, the search for each sub-path satisfying the SFC constraints is carried out sequentially using a method similar to BFS.

DQN-LBR: DQN-LBR takes into consideration the queuing delay, storage space, communication bandwidth, and propagation delay of a one-hop satellite node.

DQR: DQR is a greedy online SFC routing method based on a Deep Q Network (DQN). The difference with GDRL-SFCR is that no critic network is added, and only DQN is used as a policy network for routing decisions. The DQR algorithm only considers the remaining link bandwidth of its current node to find the next-hop node without considering the state of the neighboring nodes. The neural network parameters of the DQR algorithm are set in the same way as those of the GDRL-SFCR method.

The main reason for choosing [26,34,39] as the baseline is that [26] represents the path delay and SFC constraint optimization class of methods, but it ignores load balancing, which may lead to network congestion. Ref. [39] represents path delay and load balancing optimization class algorithms, but it does not consider SFC constraints and is suitable for fixed network function scenarios, and [34] represents load balancing and SFC constraint optimization class methods, but it ignores delay and is suitable for high-throughput but low-delay sensitive scenarios. By comparing these typical methods, the necessity of multi-objective joint optimization of path delay and load balancing in this paper can be verified.

### 5.3. Evaluation Metrics

In our experiments, we compare the performance of the above three methods regarding traffic access success rate, average network load, average end-to-end path delay, network capacity, and average running time.

- Traffic access success rate: The ratio of the amount of traffic that meets the SFC constraint transmission path to the total amount of traffic can be calculated using the following formula:
  (27) $$R_{suc}=\frac{N_{SFC}}{N_{total}}$$
  where $N_{SFC}$ represents the amount of traffic that meets the SFC constraint transmission path, and $N_{total}$ represents the total amount of traffic.
- Average network load: The load average of all satellite and ground station nodes.
- Average end-to-end path delay: The average delay of all transmission paths that meet the SFC constraint.
- Network capacity: The sum of the service rates of all transmission paths that meet the SFC constraints.
- Average running time: The ratio of the time it takes for the algorithm to complete the calculation of all traffic transmission paths to the total amount of traffic.

### 5.4. Results and Analysis

**Figure 3:**
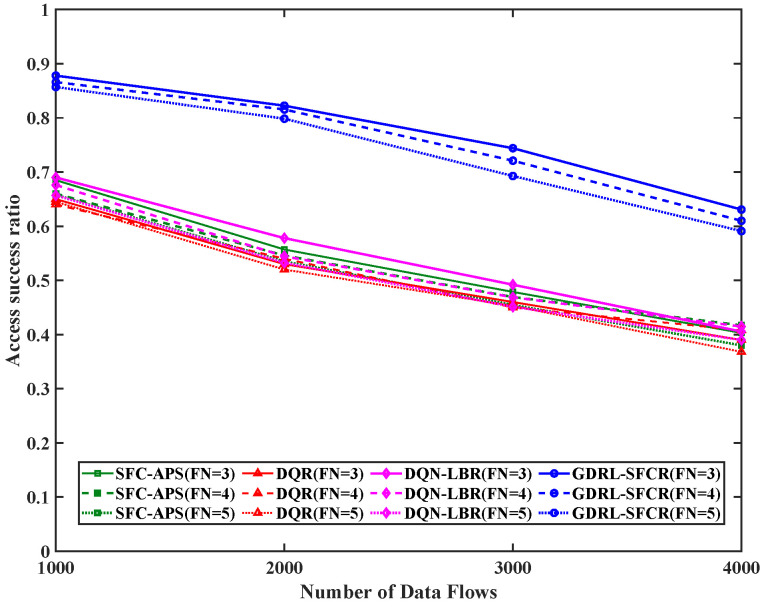
Access success rates of different data flows. The red curve is the result based on the reinforcement learning algorithm (DQR), the green curve is the result of the traditional graph theory algorithm (SFC-APS), the magenta curve is the result of the algorithm proposed in this paper (DQN-LBR), and the blue curve is the result of the algorithm proposed in this paper (GDRL-SFCR). FN represents the total number of functional nodes in the end-to-end path SFC constraint.

First, we analyzed the proposed method’s service access success rate performance. Figure 3 demonstrates the relationship between service access success rate and the number of functional constraints for different scales. As the number of function constraints increases, the access success rates of all algorithms decrease. The reason is that the increase in the number of functions in the SFC brings more constraints; thus, the number of paths satisfying the SFC constraints decreases.

In addition, as the number of data flows increases, the access success rates of all the algorithms also decrease. The reason is that the larger the data flow scale is, the greater the probability of collision during access, and the fewer the paths that satisfy the SFC conditions. Moreover, it is observed that the algorithms proposed in this paper significantly outperform the DQN-LBR, DQR and SFC-APS algorithms, and the access success rate is improved by more than 19.1%. The reason is that the SFC-APS method involves rule-based local optimization, which makes it difficult to balance global objectives in complex tasks; the DQR method does not explicitly utilize the topological relationships of graph structures, which leads to slow training of decision networks; and the DQN-LBR approach does not consider SFC constraints, which leads to some data flows failing to satisfy their SFC constraints.

**Figure 4:**
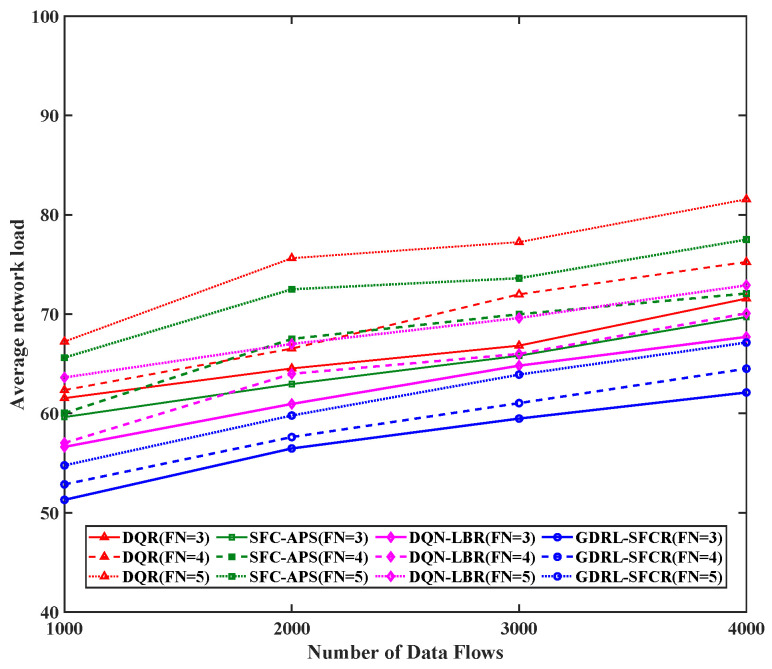
Network average load of different data flows. The red curve is the result based on the reinforcement learning algorithm (DQR), the green curve is the result of the traditional graph theory algorithm (SFC-APS), the magenta curve is the result of the algorithm proposed in this paper (DQN-LBR), and the blue curve is the result of the algorithm proposed in this paper (GDRL-SFCR). FN represents the total number of functional nodes in the end-to-end path SFC constraint.

Next, we analyzed the network average load performance of the proposed algorithm. Figure 4 shows the satellite node and ground station node average network load value with different numbers of data flows and different numbers of functional constraints. The performance of the algorithm proposed in this paper and DQN-LBR is better than that of the DQR and SFC-APS algorithms. The load decreased by more than 14.1%. The reason is that the DQR and SFC-APS methods did not consider the load of the network nodes and only used a path length similar to that of BFS as a search constraint, resulting in some nodes being fully loaded early.

**Figure 5:**
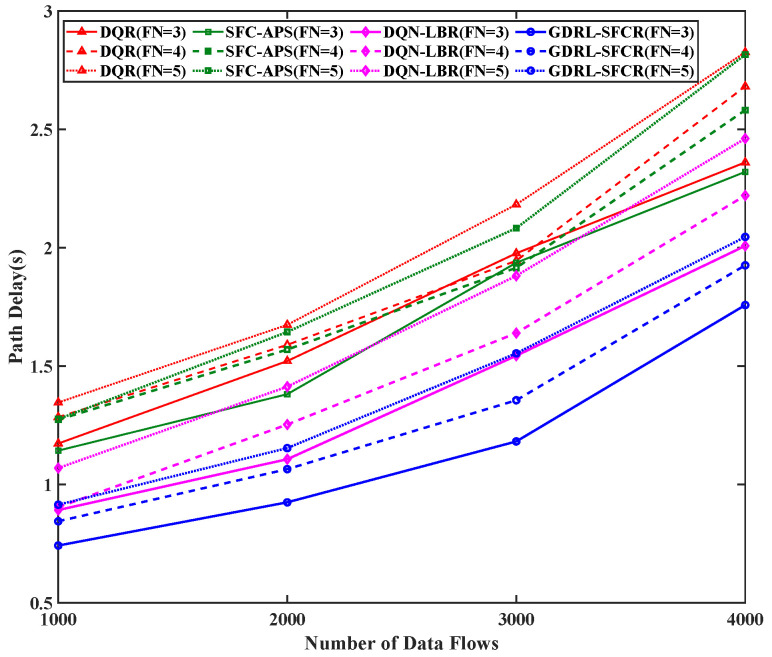
Average path delay of different data flows. The red curve is the result based on the reinforcement learning algorithm (DQR), the green curve is the result of the traditional graph theory algorithm (SFC-APS), the magenta curve is the result of the algorithm proposed in this paper (DQN-LBR), and the blue curve is the result of the algorithm proposed in this paper (GDRL-SFCR). FN represents the total number of functional nodes in the end-to-end path SFC constraint.

Then, we analyzed the path delay performance of the proposed algorithm. Figure 5 shows the average path delay under different numbers of data flows and numbers of function constraints. As seen in Figure 5, the path delay of the GDRL-SFCR algorithm is lower than other algorithms. As the number of functional constraints increases, the path delays of all the algorithms increase. However, compared to the other algorithms, the delay of the GDRL-SFCR algorithm is reduced by more than 11.3%. The reason is that the other algorithms do not consider network loading and are not sufficiently adaptive to the dynamic environment of the mega-constellation networks. When the nodes are fully loaded, the length of the searched paths that satisfy the SFC constraints increases, and the delay increases.

**Figure 6:**
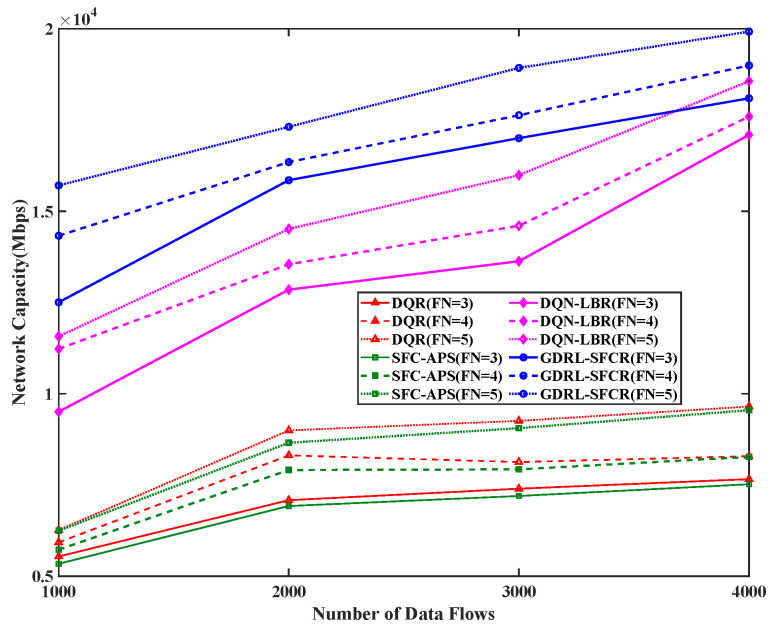
Network capacity of different data flows. The red curve is the result based on the reinforcement learning algorithm (DQR), the green curve is the result of the traditional graph theory algorithm (SFC-APS), the magenta curve is the result of the algorithm proposed in this paper (DQN-LBR), and the blue curve is the result of the algorithm proposed in this paper (GDRL-SFCR). FN represents the total number of functional nodes in the end-to-end path SFC constraint.

We also analyzed the network capacity performance of the proposed algorithm. Figure 6 shows the network capacity under different numbers of data flows and numbers of function constraints. The network capacity performance of the algorithm proposed in this paper is significantly better than that of the other algorithms. The reason is that the other algorithms do not consider the joint optimization of network load and path delay in decision making, while the GDRL-SFCR method considers the optimization of the network load, which effectively reduces the network load and increases the network capacity.

**Figure 7:**
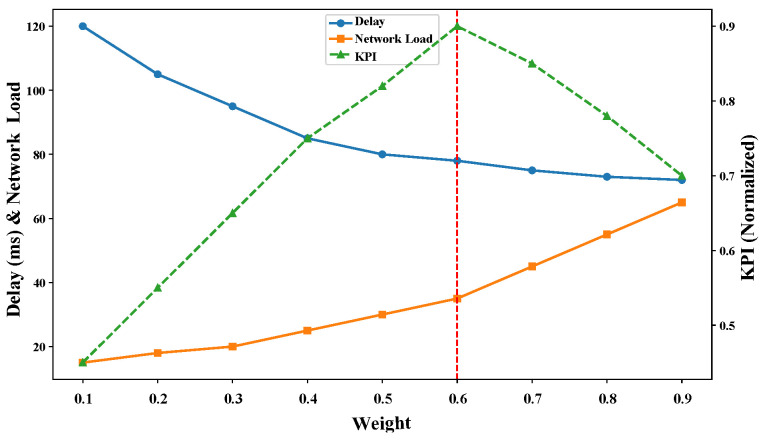
Impact of weight $\theta_{1}$ on performance metrics. The green curve is the result of KPI variation with weights. The blue curve is the path delay variance as a function of weight. The blue curve is the result of network load variance variation with weights.

We also analyzed the impact of the weights on the objectives of this paper, and we tested the delay *D*, load variance *L*, and comprehensive KPI for $\theta_{1}\in \left[0.1,0.9\right]$ (step 0.1) under the same traffic load. KPI was calculated using $KPI=\frac{1}{Delay/Delay_{max}}+\frac{1}{Load/Load_{max}}$, and $Delay_{max}$ and $Load_{max}$ are the maximum observed values in the experiment. As can be seen from Figure 7, when $\theta_{1}=0.6$ achieves a better balance between delay and load, and the algorithmic performance is insensitive to the change in $\theta_{1}$ in the interval of (0.5,0.7).

**Figure 8:**
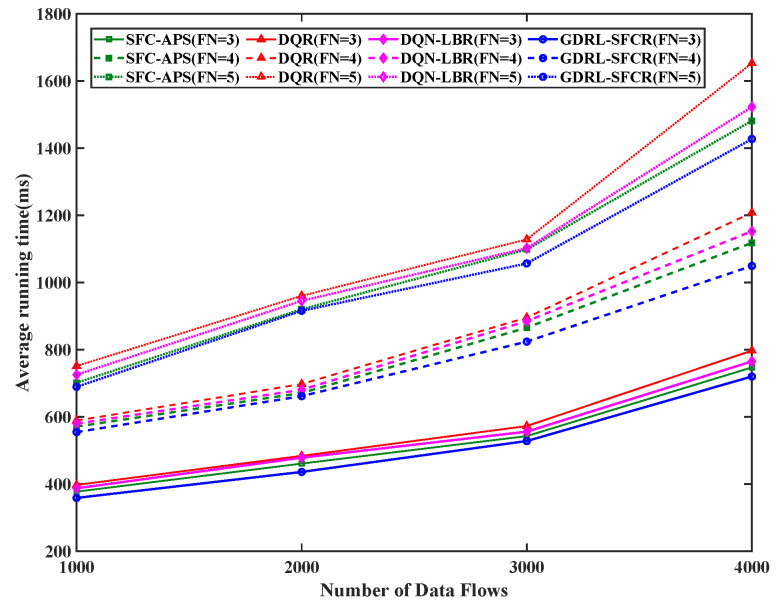
Average running time of different data flows for all algorithms. The red curve is the result based on the reinforcement learning algorithm (DQR), the green curve is the result of the traditional graph theory algorithm (SFC-APS), the magenta curve is the result of the algorithm proposed in this paper (DQN-LBR), and the blue curve is the result of the algorithm proposed in this paper (GDRL-SFCR). FN represents the total number of functional nodes in the end-to-end path SFC constraint.

Finally, we analyzed the running time performance of all the algorithms. As can be seen from Figure 8, the running time of DQR is significantly higher than thate of SFC-APS and GDRL-SFCR. In addition, when there are more than 2000 data flows, the running time of DQR increases rapidly. This is because when there are more data flows, the state space and action space in the Q-table greatly increase. In this case, finding the optimal strategy based on the table becomes difficult, as it takes more time to find the optimal strategy in large networks.

Although GDRL-SFCR takes more time to train the neural network compared to DQR, after it is trained and deployed, it simply uses the trained neural network to perform inference. The main reasons for this include high computational cost due to the complexity of updating the policy and value network, prolonged execution time due to the high-dimensional state and action space, and model scalability issues as the network size increases, leading to exponential growth in resource requirements. To address these issues, we employ several mitigating measures during training: parallelizing the task using the OpenRL [54] distributed computing framework, simplifying the dimensionality of the state space by using only the features of neighboring nodes, accelerating the training process by using GPUs, and employing optimization algorithms such as Adam to speed up convergence. These strategies can improve the efficiency and scalability of GDRL-SFCR and make it more suitable for large-scale applications.

## 6. Conclusions

In this paper, we propose the GDRL-SFCR method, which realizes end-to-end routing decision by jointly optimizing transmission delay and network load balancing while satisfying SFC constraints in low-orbit mega constellation satellite networks. The method models the routing process as an MDP and uses a graph neural network to transform node features and topological information in the satellite network into feature embeddings. It uses reinforcement learning to make routing decisions based on historical experience and the current network state, and it uses PPO to train agents to learn optimal routing policies. Extensive simulation results show that our proposed GDRL-SFCR algorithm outperforms other routing methods regarding end-to-end path delay, average network load, and service access success rate performance. Considering a real mega-constellation network scenario such as Starlink, the GNN module of the GDRL-SFCR method can be used to parse satellite orbit parameters and link status in real time, predict the dynamic connection windows of different satellites, and guide the DRL agent to plan low-latency paths in advance. Considering the computational and scalability challenges of the proposed method, it is possible to consider adding federated learning and lightweight model deployment strategies in the future to significantly reduce computational and communication overhead while ensuring routing in real-time. In addition, multi-agent joint learning is a promising approach to solving the routing decision problem of mega-constellation networks. In future work, we will consider using multi-agent reinforcement learning to study smarter routing algorithms.

## Figures and Tables

**Table 1:** Summary of existing methods for satellite routing. ✓ means that the corresponding algorithm considered the features of this column, and × means that it did not.

Work	Optimization Objective	SFC Constraints	Load Balance	Applicability Analysis
Wang et al. [27]	Max-flow	×	×	Ignore SFC constraints and load balance
Fraire et al. [28]	Path delay	×	×	Ignore SFC constraints and load balance
Zhang et al. [29,30]	Routing QoS	×	×	Ignore SFC constraints and load balance
Wu et al. [31]	Path delay	×	×	Ignore SFC constraints and load balance
Liu et al. [32]	Load balance	×	✓	Ignore SFC constraints and path delay
Yang et al. [33]	Satellites node load	×	✓	Ignore SFC constraints and path delay
Yang et al. [26]	Path delay and SFC constraints	✓	×	Ignore load balance
Zuo et al. [39]	Path delay and load balance	×	✓	Ignore SFC constraints
Xu et al. [34]	Path delay and SFC constraints	✓	×	Ignore load balance

**Table 2:** List of terms.

Term	Definition
Non-Terrestrial Networ k(NTN)	Communication networks that include satellites or other non-terrestrial nodes.
3GPP	3rd Generation Partnership Project; develops mobile communication standards.
Node Types	Satellites: Orbiting communication nodes.
	Ground Stations: Fixed communication points on Earth.
	User Terminals: Devices that connect to the network.
Links	Inter-Satellite Links (ISLs): connections between satellites.
	Ground-to-satellite links: Connections between ground stations and satellites.
	User-to-satellite links: Connections between user terminals and satellites.
Network topology	The arrangement of nodes and links in the network.
Key constraints	Link capacity: The maximum amount of data that a communication link can transmit per unit of time.
	Node capacity: Maximum amount of data that can be processed per unit of time by a satellite or ground station.
Graph Neural Network (GNN)	A type of neural network designed to operate on graph structures.
Deep Reinforcement Learning (DRL)	A machine learning technique that enables agents to learn optimal actions through trial and error.
Service Function Chaining (SFC)	A networking concept where a set of network functions is applied to a traffic flow in a specific order.

**Table 3:** Summary of simulation parameters.

Parameters	Values
Number of satellites	6048
Simulation time	5400 s
Simulation step	60 s
Satellite orbit altitude	550 km
Number of orbit planes	84
Number of satellites per plane	72
Inclination of orbit	$53^{\circ }$
Eccentricity of orbit	0
Phase factor	2
Number of ground stations	117
Number of function nodes	[3, 4, 5]
Number of user terminals	[2000, 4000, 6000, 8000]
Minimum elevation angle of ground station	$40^{\circ }$
Minimum elevation angle of user terminal	$20^{\circ }$
Central frequency	20 GHz(UL), 30 GHz(DL)
Satellite Tx gain	30 dBi
Satellite EIRP density	4 dBW/MHz
Bandwidth	250 MHz(UL), 62.5 (DL)
Satellite G/T	13 dB K^−1^
Satellite Rx gain	38.5 dBi
User terminal Rx antenna gain	39.7 dBi
User terminal noise figure	1.2 dB
User terminal antenna temperature	150 K
Ambient temperature	290 K
User terminal Tx antenna gain	43.2 dBi
User terminal Tx transmit power	2W (33 dBm)

## Data Availability

The data are contained within the article.
